# Supplementary material for: Mammary Gland Transcriptome and Proteome Modifications by Nutrient Restriction in Early Lactation Holstein Cows Challenged with Intra-Mammary Lipopolysaccharide
Source: Int J Mol Sci. 2019 Mar 6;20(5):1156. doi: 10.3390/ijms20051156 (PMC6429198; doi:10.3390/ijms20051156)
Supplement: Supplementary file 1 [file ijms-20-01156-s001.zip › ID0228_Expression Protein Details.htm]

 ID0228\_Expression Protein Details

# ID0228\_Expression Protein Details

  

## Experiment: ID0228\_CLeroux

## Report created: 10/17/2018 5:21:55 PM

## Overview image

## Data Processing Methods

|  |  |
| --- | --- |
| Peak processing method | **Centroided data; resolution = 30000 (FWHM)** |
| Peptide ion detection method | **High resolution** |

## Experiment Design

  

| Condition | Condition C | Condition R |
| --- | --- | --- |
| Replicates | 8 | 8 |

## Proteins

### Protein building options

|  |  |
| --- | --- |
| Protein grouping | **Group similar proteins** |
| Protein quantitation | **Relative Quantitation using Hi-5** |

  

| Accession | Peptides | Score | Anova (p)\* | Fold | Tags | Description | Average Normalised Abundances | |
| --- | --- | --- | --- | --- | --- | --- | --- | --- |
|  |  |  |  |  |  |  | Condition C | Condition R |
| 1::HBB\_BOVIN | 18 (18) | 1356.29 | 0.03 | 1.30 |  | Hemoglobin subunit beta OS=Bos taurus GN=HBB PE=1 SV=1 | 5.83e+008 | 7.58e+008 |
| 1::PDIA3\_BOVIN | 16 (16) | 845.09 | 0.09 | 1.11 |  | Protein disulfide-isomerase A3 OS=Bos taurus GN=PDIA3 PE=2 SV=1 | 3.79e+007 | 3.41e+007 |
| 2::Q1RMN8 | 5 (5) | 543.36 | 0.02 | 1.89 |  | TREMBL:Q1RMN8 (Bos taurus) Similar to Immunoglobulin lambda-like polypeptide 1 | 2.03e+008 | 3.84e+008 |
| 1::F1MH40\_BOVIN | 7 (7) | 542.47 | 0.04 | 1.50 |  | Uncharacterized protein OS=Bos taurus PE=1 SV=2 | 2.69e+007 | 4.03e+007 |
| 1::E1BF20\_BOVIN | 8 (8) | 518.75 | 9.96e-003 | 1.24 |  | Uncharacterized protein OS=Bos taurus GN=HNRNPH1 PE=4 SV=2 | 1.35e+007 | 1.08e+007 |
| 1::TCPD\_BOVIN | 9 (9) | 512.81 | 0.02 | 1.25 |  | T-complex protein 1 subunit delta OS=Bos taurus GN=CCT4 PE=1 SV=3 | 6.54e+006 | 5.23e+006 |
| 1::A7E307\_BOVIN | 10 (10) | 478.07 | 0.23 | 1.08 |  | DDX17 protein OS=Bos taurus GN=DDX17 PE=1 SV=1 | 8.14e+006 | 7.54e+006 |
| 1::PDIA4\_BOVIN | 10 (10) | 441.10 | 0.02 | 1.20 |  | Protein disulfide-isomerase A4 OS=Bos taurus GN=PDIA4 PE=2 SV=1 | 8.12e+006 | 6.76e+006 |
| 1::HBA\_BOVIN | 8 (8) | 408.92 | 0.04 | 1.31 |  | Hemoglobin subunit alpha OS=Bos taurus GN=HBA PE=1 SV=2 | 3.28e+008 | 4.30e+008 |
| 1::F6PWD5\_BOVIN | 6 (6) | 396.20 | 0.03 | 1.29 |  | Uncharacterized protein (Fragment) OS=Bos taurus PE=4 SV=1 | 2.09e+007 | 1.62e+007 |
| 1::RS27A\_BOVIN | 6 (6) | 387.82 | 0.04 | 1.19 |  | Ubiquitin-40S ribosomal protein S27a OS=Bos taurus GN=RPS27A PE=1 SV=2 | 7.06e+007 | 5.92e+007 |
| 1::F1N6Y1\_BOVIN | 9 (9) | 378.04 | 0.02 | 1.23 |  | Uncharacterized protein OS=Bos taurus GN=GANAB PE=1 SV=1 | 7.16e+006 | 5.83e+006 |
| 1::DHX9\_BOVIN | 9 (9) | 359.62 | 0.02 | 1.31 |  | ATP-dependent RNA helicase A OS=Bos taurus GN=DHX9 PE=2 SV=1 | 7.46e+006 | 5.70e+006 |
| 1::RPN2\_BOVIN | 6 (6) | 351.38 | 0.03 | 1.50 |  | Dolichyl-diphosphooligosaccharide--protein glycosyltransferase subunit 2 OS=Bos taurus GN=RPN2 PE=2 SV=1 | 2.80e+006 | 1.86e+006 |
| 1::E1BEG2\_BOVIN | 4 (4) | 279.23 | 0.03 | 1.15 |  | Uncharacterized protein OS=Bos taurus GN=HNRNPA3 PE=1 SV=2 | 1.27e+007 | 1.11e+007 |
| 1::NACA\_BOVIN | 4 (4) | 274.55 | 0.05 | 1.21 |  | Nascent polypeptide-associated complex subunit alpha OS=Bos taurus GN=NACA PE=1 SV=1 | 7.72e+006 | 6.40e+006 |
| 1::ARPC2\_BOVIN | 7 (7) | 270.30 | 0.05 | 1.11 |  | Actin-related protein 2/3 complex subunit 2 OS=Bos taurus GN=ARPC2 PE=1 SV=1 | 1.29e+007 | 1.44e+007 |
| 1::PPIB\_BOVIN | 4 (4) | 258.86 | 4.23e-003 | 1.36 |  | Peptidyl-prolyl cis-trans isomerase B OS=Bos taurus GN=PPIB PE=1 SV=4 | 2.28e+007 | 1.68e+007 |
| 1::SPA35\_BOVIN | 3 (3) | 256.91 | 0.03 | 1.99 |  | Serpin A3-5 OS=Bos taurus GN=SERPINA3-5 PE=3 SV=1 | 2.15e+007 | 4.29e+007 |
| 1::RS2\_BOVIN | 5 (5) | 240.00 | 0.03 | 1.21 |  | 40S ribosomal protein S2 OS=Bos taurus GN=RPS2 PE=2 SV=2 | 1.49e+007 | 1.23e+007 |
| 1::G8JKW7\_BOVIN | 3 (3) | 231.21 | 0.02 | 2.40 |  | Uncharacterized protein OS=Bos taurus GN=SERPINA3 PE=3 SV=1 | 5.94e+006 | 1.43e+007 |
| 1::GBB1\_BOVIN | 4 (4) | 226.38 | 0.02 | 1.26 |  | Guanine nucleotide-binding protein G(I)/G(S)/G(T) subunit beta-1 OS=Bos taurus GN=GNB1 PE=1 SV=3 | 6.17e+006 | 4.90e+006 |
| 1::YBOX1\_BOVIN | 5 (5) | 214.76 | 3.86e-003 | 1.73 |  | Nuclease-sensitive element-binding protein 1 OS=Bos taurus GN=YBX1 PE=2 SV=3 | 2.15e+006 | 1.24e+006 |
| 1::RS15\_BOVIN | 3 (3) | 209.88 | 0.03 | 1.40 |  | 40S ribosomal protein S15 OS=Bos taurus GN=RPS15 PE=2 SV=3 | 2.57e+007 | 1.83e+007 |
| 1::E1BBY7\_BOVIN | 5 (5) | 206.71 | 1.83e-003 | 1.45 |  | Uncharacterized protein OS=Bos taurus GN=HSPA4 PE=1 SV=2 | 1.91e+006 | 1.32e+006 |
| 1::F1MLW8\_BOVIN | 3 (3) | 206.33 | 4.75e-003 | 5.49 |  | Uncharacterized protein OS=Bos taurus PE=4 SV=2 | 3.17e+006 | 1.74e+007 |
| 1::Q3SYT9\_BOVIN | 3 (3) | 177.92 | 9.31e-003 | 1.18 |  | Poly(RC) binding protein 2 OS=Bos taurus GN=PCBP2 PE=1 SV=1 | 6.71e+006 | 7.91e+006 |
| 1::PP2AA\_BOVIN | 4 (4) | 155.92 | 9.97e-003 | 1.19 |  | Serine/threonine-protein phosphatase 2A catalytic subunit alpha isoform OS=Bos taurus GN=PPP2CA PE=1 SV=1 | 6.34e+006 | 5.32e+006 |
| 1::RL10\_BOVIN | 3 (3) | 153.13 | 0.03 | 1.86 |  | 60S ribosomal protein L10 OS=Bos taurus GN=RPL10 PE=2 SV=4 | 4.93e+006 | 2.65e+006 |
| 1::G3MY19\_BOVIN | 3 (3) | 151.45 | 0.04 | 1.80 |  | Uncharacterized protein OS=Bos taurus GN=PDLIM5 PE=4 SV=1 | 1.54e+006 | 8.60e+005 |
| 1::A3KN04\_BOVIN | 3 (3) | 148.24 | 0.03 | 1.31 |  | Dolichyl-diphosphooligosaccharide--protein glycosyltransferase subunit 1 OS=Bos taurus GN=RPN1 PE=1 SV=1 | 6.46e+006 | 4.93e+006 |
| 1::Q3SX47\_BOVIN | 2 (2) | 146.99 | 0.02 | 1.22 |  | Heterogeneous nuclear ribonucleoprotein C (C1/C2) OS=Bos taurus GN=HNRPC PE=2 SV=1 | 5.22e+006 | 4.30e+006 |
| 1::A6QP36\_BOVIN | 4 (4) | 140.38 | 0.02 | 1.22 |  | LMAN2 protein OS=Bos taurus GN=LMAN2 PE=2 SV=1 | 4.37e+006 | 3.57e+006 |
| 1::PSA3\_BOVIN | 2 (2) | 128.32 | 0.04 | 1.21 |  | Proteasome subunit alpha type-3 OS=Bos taurus GN=PSMA3 PE=1 SV=3 | 7.93e+006 | 6.57e+006 |
| 1::CAZA2\_BOVIN | 4 (4) | 124.84 | 0.02 | 1.39 |  | F-actin-capping protein subunit alpha-2 OS=Bos taurus GN=CAPZA2 PE=1 SV=3 | 3.67e+006 | 2.64e+006 |
| 1::Q2KJH7\_BOVIN | 3 (3) | 121.89 | 0.02 | 1.73 |  | Aldehyde dehydrogenase 18 family, member A1 OS=Bos taurus GN=ALDH18A1 PE=2 SV=1 | 3.41e+007 | 5.88e+007 |
| 1::PSMD2\_BOVIN | 4 (4) | 121.33 | 2.73e-003 | 1.37 |  | 26S proteasome non-ATPase regulatory subunit 2 OS=Bos taurus GN=PSMD2 PE=1 SV=2 | 4.60e+006 | 3.36e+006 |
| 1::PRDBP\_BOVIN | 2 (2) | 102.84 | 0.03 | 1.14 |  | Protein kinase C delta-binding protein OS=Bos taurus GN=PRKCDBP PE=2 SV=1 | 1.08e+007 | 9.48e+006 |
| 1::A6H7H3\_BOVIN | 2 (2) | 100.91 | 2.14e-003 | 2.54 |  | LOC789567 protein OS=Bos taurus GN=LOC789567 PE=2 SV=1 | 2.09e+006 | 8.23e+005 |
| 1::F2Z4F0\_BOVIN | 4 (4) | 97.27 | 0.03 | 1.34 |  | Uncharacterized protein OS=Bos taurus GN=ACTR1A PE=1 SV=1 | 2.13e+006 | 1.59e+006 |
| 1::F1N1G7\_BOVIN | 2 (2) | 91.70 | 0.01 | 1.28 |  | Kinesin-like protein OS=Bos taurus GN=KIF5B PE=1 SV=2 | 1.96e+006 | 1.53e+006 |
| 1::STA5A\_BOVIN | 2 (2) | 87.68 | 0.05 | 1.39 |  | Signal transducer and activator of transcription 5A OS=Bos taurus GN=STAT5A PE=2 SV=2 | 2.20e+006 | 1.58e+006 |
| 1::CASP6\_BOVIN | 2 (2) | 86.12 | 0.03 | 1.47 |  | Caspase-6 OS=Bos taurus GN=CASP6 PE=2 SV=1 | 2.86e+006 | 1.94e+006 |
| 1::A8E4P2\_BOVIN | 3 (3) | 85.93 | 0.04 | 1.42 |  | FARSB protein OS=Bos taurus GN=FARSB PE=2 SV=1 | 4.20e+006 | 2.97e+006 |
| 1::F1MN61\_BOVIN | 2 (2) | 85.75 | 0.04 | 2.00 |  | Uncharacterized protein (Fragment) OS=Bos taurus GN=EEA1 PE=1 SV=2 | 1.09e+006 | 5.46e+005 |
| 1::ARC1B\_BOVIN | 2 (2) | 80.22 | 9.58e-003 | 2.20 |  | Actin-related protein 2/3 complex subunit 1B OS=Bos taurus GN=ARPC1B PE=1 SV=4 | 3.30e+006 | 1.50e+006 |
| 1::A6H788\_BOVIN | 2 (2) | 79.16 | 0.02 | 1.29 |  | SNRPA1 protein OS=Bos taurus GN=SNRPA1 PE=2 SV=1 | 1.78e+006 | 1.37e+006 |
| 1::Q3T0J0\_BOVIN | 2 (2) | 77.32 | 0.03 | 1.37 |  | 3'-phosphoadenosine 5'-phosphosulfate synthase 1 OS=Bos taurus GN=PAPSS1 PE=2 SV=1 | 3.43e+006 | 2.50e+006 |
| 1::EIF3H\_BOVIN | 2 (2) | 75.15 | 5.32e-003 | 1.68 |  | Eukaryotic translation initiation factor 3 subunit H OS=Bos taurus GN=EIF3H PE=2 SV=1 | 2.26e+006 | 1.34e+006 |
| 1::E1BKX3\_BOVIN | 3 (3) | 75.11 | 0.02 | 1.94 |  | Uncharacterized protein OS=Bos taurus GN=MYBBP1A PE=1 SV=1 | 1.96e+006 | 1.01e+006 |
| 1::F6QE33\_BOVIN | 2 (2) | 73.82 | 0.03 | 1.84 |  | Uncharacterized protein OS=Bos taurus GN=COPS7A PE=4 SV=1 | 1.59e+006 | 8.63e+005 |
| 1::G3X6L8\_BOVIN | 2 (2) | 60.63 | 0.02 | 1.60 |  | Uncharacterized protein OS=Bos taurus GN=NIPSNAP3A PE=4 SV=1 | 1.57e+006 | 9.79e+005 |
| 1::PLBL2\_BOVIN | 2 (2) | 39.97 | 9.21e-003 | 1.26 |  | Putative phospholipase B-like 2 OS=Bos taurus GN=PLBD2 PE=2 SV=1 | 1.99e+006 | 1.59e+006 |

  

| Tags | |
| --- | --- |
|  | Anova p-value ≤ 0.05 |
|  | M |
|  | B |
|  | O |
|  | 1 seul peptide |

## Accession 1::E1BBY7\_BOVIN

  

|  |  |
| --- | --- |
| Description | Uncharacterized protein OS=Bos taurus GN=HSPA4 PE=1 SV=2 |
| Peptides | 5 (5) |
| Score | 206.71 |
| Anova | 1.83e-003 |
| Fold | 1.45 |

## Accession 1::A6H7H3\_BOVIN

  

|  |  |
| --- | --- |
| Description | LOC789567 protein OS=Bos taurus GN=LOC789567 PE=2 SV=1 |
| Peptides | 2 (2) |
| Score | 100.91 |
| Anova | 2.14e-003 |
| Fold | 2.54 |

## Accession 1::PSMD2\_BOVIN

  

|  |  |
| --- | --- |
| Description | 26S proteasome non-ATPase regulatory subunit 2 OS=Bos taurus GN=PSMD2 PE=1 SV=2 |
| Peptides | 4 (4) |
| Score | 121.33 |
| Anova | 2.73e-003 |
| Fold | 1.37 |

## Accession 1::YBOX1\_BOVIN (+1)

  

|  |  |
| --- | --- |
| Description | Nuclease-sensitive element-binding protein 1 OS=Bos taurus GN=YBX1 PE=2 SV=3 |
| Peptides | 5 (5) |
| Score | 214.76 |
| Anova | 3.86e-003 |
| Fold | 1.73 |

## Accession 1::PPIB\_BOVIN (+1)

  

|  |  |
| --- | --- |
| Description | Peptidyl-prolyl cis-trans isomerase B OS=Bos taurus GN=PPIB PE=1 SV=4 |
| Peptides | 4 (4) |
| Score | 258.86 |
| Anova | 4.23e-003 |
| Fold | 1.36 |

## Accession 1::F1MLW8\_BOVIN

  

|  |  |
| --- | --- |
| Description | Uncharacterized protein OS=Bos taurus PE=4 SV=2 |
| Peptides | 3 (3) |
| Score | 206.33 |
| Anova | 4.75e-003 |
| Fold | 5.49 |

## Accession 1::EIF3H\_BOVIN

  

|  |  |
| --- | --- |
| Description | Eukaryotic translation initiation factor 3 subunit H OS=Bos taurus GN=EIF3H PE=2 SV=1 |
| Peptides | 2 (2) |
| Score | 75.15 |
| Anova | 5.32e-003 |
| Fold | 1.68 |

## Accession 1::PLBL2\_BOVIN

  

|  |  |
| --- | --- |
| Description | Putative phospholipase B-like 2 OS=Bos taurus GN=PLBD2 PE=2 SV=1 |
| Peptides | 2 (2) |
| Score | 39.97 |
| Anova | 9.21e-003 |
| Fold | 1.26 |

## Accession 1::Q3SYT9\_BOVIN

  

|  |  |
| --- | --- |
| Description | Poly(RC) binding protein 2 OS=Bos taurus GN=PCBP2 PE=1 SV=1 |
| Peptides | 3 (3) |
| Score | 177.92 |
| Anova | 9.31e-003 |
| Fold | 1.18 |

## Accession 1::ARC1B\_BOVIN

  

|  |  |
| --- | --- |
| Description | Actin-related protein 2/3 complex subunit 1B OS=Bos taurus GN=ARPC1B PE=1 SV=4 |
| Peptides | 2 (2) |
| Score | 80.22 |
| Anova | 9.58e-003 |
| Fold | 2.20 |

## Accession 1::E1BF20\_BOVIN (+2)

  

|  |  |
| --- | --- |
| Description | Uncharacterized protein OS=Bos taurus GN=HNRNPH1 PE=4 SV=2 |
| Peptides | 8 (8) |
| Score | 518.75 |
| Anova | 9.96e-003 |
| Fold | 1.24 |

## Accession 1::PP2AA\_BOVIN

  

|  |  |
| --- | --- |
| Description | Serine/threonine-protein phosphatase 2A catalytic subunit alpha isoform OS=Bos taurus GN=PPP2CA PE=1 SV=1 |
| Peptides | 4 (4) |
| Score | 155.92 |
| Anova | 9.97e-003 |
| Fold | 1.19 |

## Accession 1::F1N1G7\_BOVIN

  

|  |  |
| --- | --- |
| Description | Kinesin-like protein OS=Bos taurus GN=KIF5B PE=1 SV=2 |
| Peptides | 2 (2) |
| Score | 91.70 |
| Anova | 0.01 |
| Fold | 1.28 |

## Accession 1::GBB1\_BOVIN

  

|  |  |
| --- | --- |
| Description | Guanine nucleotide-binding protein G(I)/G(S)/G(T) subunit beta-1 OS=Bos taurus GN=GNB1 PE=1 SV=3 |
| Peptides | 4 (4) |
| Score | 226.38 |
| Anova | 0.02 |
| Fold | 1.26 |

## Accession 1::G3X6L8\_BOVIN

  

|  |  |
| --- | --- |
| Description | Uncharacterized protein OS=Bos taurus GN=NIPSNAP3A PE=4 SV=1 |
| Peptides | 2 (2) |
| Score | 60.63 |
| Anova | 0.02 |
| Fold | 1.60 |

## Accession 1::Q2KJH7\_BOVIN

  

|  |  |
| --- | --- |
| Description | Aldehyde dehydrogenase 18 family, member A1 OS=Bos taurus GN=ALDH18A1 PE=2 SV=1 |
| Peptides | 3 (3) |
| Score | 121.89 |
| Anova | 0.02 |
| Fold | 1.73 |

## Accession 1::A6QP36\_BOVIN

  

|  |  |
| --- | --- |
| Description | LMAN2 protein OS=Bos taurus GN=LMAN2 PE=2 SV=1 |
| Peptides | 4 (4) |
| Score | 140.38 |
| Anova | 0.02 |
| Fold | 1.22 |

## Accession 1::DHX9\_BOVIN

  

|  |  |
| --- | --- |
| Description | ATP-dependent RNA helicase A OS=Bos taurus GN=DHX9 PE=2 SV=1 |
| Peptides | 9 (9) |
| Score | 359.62 |
| Anova | 0.02 |
| Fold | 1.31 |

## Accession 1::Q3SX47\_BOVIN

  

|  |  |
| --- | --- |
| Description | Heterogeneous nuclear ribonucleoprotein C (C1/C2) OS=Bos taurus GN=HNRPC PE=2 SV=1 |
| Peptides | 2 (2) |
| Score | 146.99 |
| Anova | 0.02 |
| Fold | 1.22 |

## Accession 1::A6H788\_BOVIN

  

|  |  |
| --- | --- |
| Description | SNRPA1 protein OS=Bos taurus GN=SNRPA1 PE=2 SV=1 |
| Peptides | 2 (2) |
| Score | 79.16 |
| Anova | 0.02 |
| Fold | 1.29 |

## Accession 1::F1N6Y1\_BOVIN

  

|  |  |
| --- | --- |
| Description | Uncharacterized protein OS=Bos taurus GN=GANAB PE=1 SV=1 |
| Peptides | 9 (9) |
| Score | 378.04 |
| Anova | 0.02 |
| Fold | 1.23 |

## Accession 1::E1BKX3\_BOVIN

  

|  |  |
| --- | --- |
| Description | Uncharacterized protein OS=Bos taurus GN=MYBBP1A PE=1 SV=1 |
| Peptides | 3 (3) |
| Score | 75.11 |
| Anova | 0.02 |
| Fold | 1.94 |

## Accession 1::TCPD\_BOVIN

  

|  |  |
| --- | --- |
| Description | T-complex protein 1 subunit delta OS=Bos taurus GN=CCT4 PE=1 SV=3 |
| Peptides | 9 (9) |
| Score | 512.81 |
| Anova | 0.02 |
| Fold | 1.25 |

## Accession 1::G8JKW7\_BOVIN

  

|  |  |
| --- | --- |
| Description | Uncharacterized protein OS=Bos taurus GN=SERPINA3 PE=3 SV=1 |
| Peptides | 3 (3) |
| Score | 231.21 |
| Anova | 0.02 |
| Fold | 2.40 |

## Accession 1::CAZA2\_BOVIN

  

|  |  |
| --- | --- |
| Description | F-actin-capping protein subunit alpha-2 OS=Bos taurus GN=CAPZA2 PE=1 SV=3 |
| Peptides | 4 (4) |
| Score | 124.84 |
| Anova | 0.02 |
| Fold | 1.39 |

## Accession 2::Q1RMN8 (+1)

  

|  |  |
| --- | --- |
| Description | TREMBL:Q1RMN8 (Bos taurus) Similar to Immunoglobulin lambda-like polypeptide 1 |
| Peptides | 5 (5) |
| Score | 543.36 |
| Anova | 0.02 |
| Fold | 1.89 |

## Accession 1::PDIA4\_BOVIN

  

|  |  |
| --- | --- |
| Description | Protein disulfide-isomerase A4 OS=Bos taurus GN=PDIA4 PE=2 SV=1 |
| Peptides | 10 (10) |
| Score | 441.10 |
| Anova | 0.02 |
| Fold | 1.20 |

## Accession 1::F6PWD5\_BOVIN (+3)

  

|  |  |
| --- | --- |
| Description | Uncharacterized protein (Fragment) OS=Bos taurus PE=4 SV=1 |
| Peptides | 6 (6) |
| Score | 396.20 |
| Anova | 0.03 |
| Fold | 1.29 |

## Accession 1::E1BEG2\_BOVIN (+1)

  

|  |  |
| --- | --- |
| Description | Uncharacterized protein OS=Bos taurus GN=HNRNPA3 PE=1 SV=2 |
| Peptides | 4 (4) |
| Score | 279.23 |
| Anova | 0.03 |
| Fold | 1.15 |

## Accession 1::F6QE33\_BOVIN

  

|  |  |
| --- | --- |
| Description | Uncharacterized protein OS=Bos taurus GN=COPS7A PE=4 SV=1 |
| Peptides | 2 (2) |
| Score | 73.82 |
| Anova | 0.03 |
| Fold | 1.84 |

## Accession 1::SPA35\_BOVIN

  

|  |  |
| --- | --- |
| Description | Serpin A3-5 OS=Bos taurus GN=SERPINA3-5 PE=3 SV=1 |
| Peptides | 3 (3) |
| Score | 256.91 |
| Anova | 0.03 |
| Fold | 1.99 |

## Accession 1::RPN2\_BOVIN

  

|  |  |
| --- | --- |
| Description | Dolichyl-diphosphooligosaccharide--protein glycosyltransferase subunit 2 OS=Bos taurus GN=RPN2 PE=2 SV=1 |
| Peptides | 6 (6) |
| Score | 351.38 |
| Anova | 0.03 |
| Fold | 1.50 |

## Accession 1::A3KN04\_BOVIN

  

|  |  |
| --- | --- |
| Description | Dolichyl-diphosphooligosaccharide--protein glycosyltransferase subunit 1 OS=Bos taurus GN=RPN1 PE=1 SV=1 |
| Peptides | 3 (3) |
| Score | 148.24 |
| Anova | 0.03 |
| Fold | 1.31 |

## Accession 1::CASP6\_BOVIN

  

|  |  |
| --- | --- |
| Description | Caspase-6 OS=Bos taurus GN=CASP6 PE=2 SV=1 |
| Peptides | 2 (2) |
| Score | 86.12 |
| Anova | 0.03 |
| Fold | 1.47 |

## Accession 1::RS2\_BOVIN

  

|  |  |
| --- | --- |
| Description | 40S ribosomal protein S2 OS=Bos taurus GN=RPS2 PE=2 SV=2 |
| Peptides | 5 (5) |
| Score | 240.00 |
| Anova | 0.03 |
| Fold | 1.21 |

## Accession 1::Q3T0J0\_BOVIN

  

|  |  |
| --- | --- |
| Description | 3'-phosphoadenosine 5'-phosphosulfate synthase 1 OS=Bos taurus GN=PAPSS1 PE=2 SV=1 |
| Peptides | 2 (2) |
| Score | 77.32 |
| Anova | 0.03 |
| Fold | 1.37 |

## Accession 1::HBB\_BOVIN (+4)

  

|  |  |
| --- | --- |
| Description | Hemoglobin subunit beta OS=Bos taurus GN=HBB PE=1 SV=1 |
| Peptides | 18 (18) |
| Score | 1356.29 |
| Anova | 0.03 |
| Fold | 1.30 |

## Accession 1::RL10\_BOVIN (+2)

  

|  |  |
| --- | --- |
| Description | 60S ribosomal protein L10 OS=Bos taurus GN=RPL10 PE=2 SV=4 |
| Peptides | 3 (3) |
| Score | 153.13 |
| Anova | 0.03 |
| Fold | 1.86 |

## Accession 1::F2Z4F0\_BOVIN

  

|  |  |
| --- | --- |
| Description | Uncharacterized protein OS=Bos taurus GN=ACTR1A PE=1 SV=1 |
| Peptides | 4 (4) |
| Score | 97.27 |
| Anova | 0.03 |
| Fold | 1.34 |

## Accession 1::RS15\_BOVIN

  

|  |  |
| --- | --- |
| Description | 40S ribosomal protein S15 OS=Bos taurus GN=RPS15 PE=2 SV=3 |
| Peptides | 3 (3) |
| Score | 209.88 |
| Anova | 0.03 |
| Fold | 1.40 |

## Accession 1::PRDBP\_BOVIN

  

|  |  |
| --- | --- |
| Description | Protein kinase C delta-binding protein OS=Bos taurus GN=PRKCDBP PE=2 SV=1 |
| Peptides | 2 (2) |
| Score | 102.84 |
| Anova | 0.03 |
| Fold | 1.14 |

## Accession 1::G3MY19\_BOVIN

  

|  |  |
| --- | --- |
| Description | Uncharacterized protein OS=Bos taurus GN=PDLIM5 PE=4 SV=1 |
| Peptides | 3 (3) |
| Score | 151.45 |
| Anova | 0.04 |
| Fold | 1.80 |

## Accession 1::F1MH40\_BOVIN

  

|  |  |
| --- | --- |
| Description | Uncharacterized protein OS=Bos taurus PE=1 SV=2 |
| Peptides | 7 (7) |
| Score | 542.47 |
| Anova | 0.04 |
| Fold | 1.50 |

## Accession 1::PSA3\_BOVIN

  

|  |  |
| --- | --- |
| Description | Proteasome subunit alpha type-3 OS=Bos taurus GN=PSMA3 PE=1 SV=3 |
| Peptides | 2 (2) |
| Score | 128.32 |
| Anova | 0.04 |
| Fold | 1.21 |

## Accession 1::RS27A\_BOVIN (+1)

  

|  |  |
| --- | --- |
| Description | Ubiquitin-40S ribosomal protein S27a OS=Bos taurus GN=RPS27A PE=1 SV=2 |
| Peptides | 6 (6) |
| Score | 387.82 |
| Anova | 0.04 |
| Fold | 1.19 |

## Accession 1::A8E4P2\_BOVIN

  

|  |  |
| --- | --- |
| Description | FARSB protein OS=Bos taurus GN=FARSB PE=2 SV=1 |
| Peptides | 3 (3) |
| Score | 85.93 |
| Anova | 0.04 |
| Fold | 1.42 |

## Accession 1::HBA\_BOVIN (+1)

  

|  |  |
| --- | --- |
| Description | Hemoglobin subunit alpha OS=Bos taurus GN=HBA PE=1 SV=2 |
| Peptides | 8 (8) |
| Score | 408.92 |
| Anova | 0.04 |
| Fold | 1.31 |

## Accession 1::F1MN61\_BOVIN

  

|  |  |
| --- | --- |
| Description | Uncharacterized protein (Fragment) OS=Bos taurus GN=EEA1 PE=1 SV=2 |
| Peptides | 2 (2) |
| Score | 85.75 |
| Anova | 0.04 |
| Fold | 2.00 |

## Accession 1::NACA\_BOVIN

  

|  |  |
| --- | --- |
| Description | Nascent polypeptide-associated complex subunit alpha OS=Bos taurus GN=NACA PE=1 SV=1 |
| Peptides | 4 (4) |
| Score | 274.55 |
| Anova | 0.05 |
| Fold | 1.21 |

## Accession 1::STA5A\_BOVIN

  

|  |  |
| --- | --- |
| Description | Signal transducer and activator of transcription 5A OS=Bos taurus GN=STAT5A PE=2 SV=2 |
| Peptides | 2 (2) |
| Score | 87.68 |
| Anova | 0.05 |
| Fold | 1.39 |

## Accession 1::ARPC2\_BOVIN

  

|  |  |
| --- | --- |
| Description | Actin-related protein 2/3 complex subunit 2 OS=Bos taurus GN=ARPC2 PE=1 SV=1 |
| Peptides | 7 (7) |
| Score | 270.30 |
| Anova | 0.05 |
| Fold | 1.11 |

## Accession 1::PDIA3\_BOVIN

  

|  |  |
| --- | --- |
| Description | Protein disulfide-isomerase A3 OS=Bos taurus GN=PDIA3 PE=2 SV=1 |
| Peptides | 16 (16) |
| Score | 845.09 |
| Anova | 0.09 |
| Fold | 1.11 |

## Accession 1::A7E307\_BOVIN (+1)

  

|  |  |
| --- | --- |
| Description | DDX17 protein OS=Bos taurus GN=DDX17 PE=1 SV=1 |
| Peptides | 10 (10) |
| Score | 478.07 |
| Anova | 0.23 |
| Fold | 1.08 |
