# Supplementary figures and images for: Mammary Gland Transcriptome and Proteome Modifications by Nutrient Restriction in Early Lactation Holstein Cows Challenged with Intra-Mammary Lipopolysaccharide
Source: Int J Mol Sci. 2019 Mar 6;20(5):1156. doi: 10.3390/ijms20051156 (PMC6429198; doi:10.3390/ijms20051156)

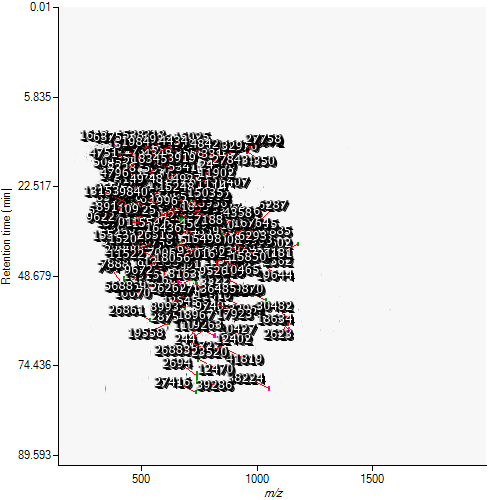

Supplement: Supplementary file 1 [file ijms-20-01156-s001.zip › ID0228_Expression Protein Details_files/class%20ID0228_CLeroux.png]

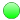

Supplement: Supplementary file 1 [file ijms-20-01156-s001.zip › ID0228_Expression Protein Details_files/p_category0.png]

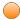

Supplement: Supplementary file 1 [file ijms-20-01156-s001.zip › ID0228_Expression Protein Details_files/p_category1.png]

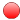

Supplement: Supplementary file 1 [file ijms-20-01156-s001.zip › ID0228_Expression Protein Details_files/p_category2.png]

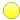

Supplement: Supplementary file 1 [file ijms-20-01156-s001.zip › ID0228_Expression Protein Details_files/p_category3.png]

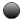

Supplement: Supplementary file 1 [file ijms-20-01156-s001.zip › ID0228_Expression Protein Details_files/p_category4.png]

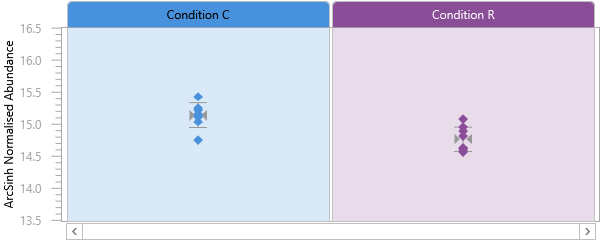

Supplement: Supplementary file 1 [file ijms-20-01156-s001.zip › ID0228_Expression Protein Details_files/protein1_graph.png]

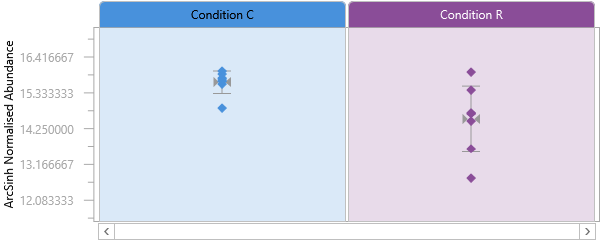

Supplement: Supplementary file 1 [file ijms-20-01156-s001.zip › ID0228_Expression Protein Details_files/protein10_graph.png]

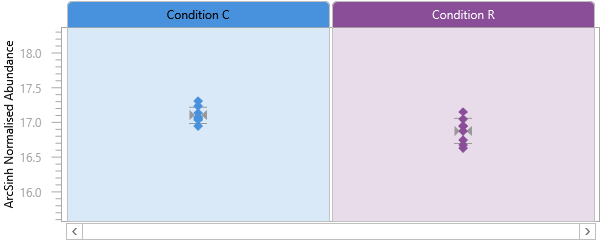

Supplement: Supplementary file 1 [file ijms-20-01156-s001.zip › ID0228_Expression Protein Details_files/protein11_graph.png]

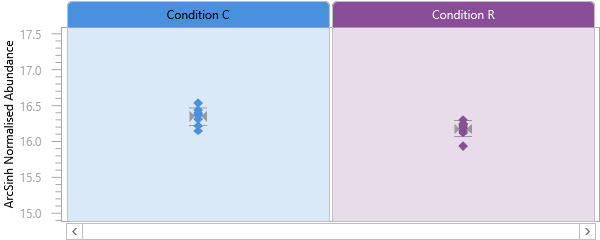

Supplement: Supplementary file 1 [file ijms-20-01156-s001.zip › ID0228_Expression Protein Details_files/protein12_graph.png]

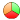

Supplement: Supplementary file 1 [file ijms-20-01156-s001.zip › ID0228_Expression Protein Details_files/protein12Category.png]

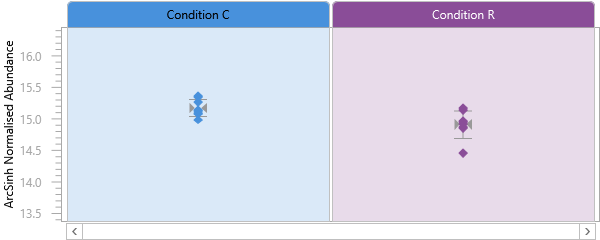

Supplement: Supplementary file 1 [file ijms-20-01156-s001.zip › ID0228_Expression Protein Details_files/protein13_graph.png]

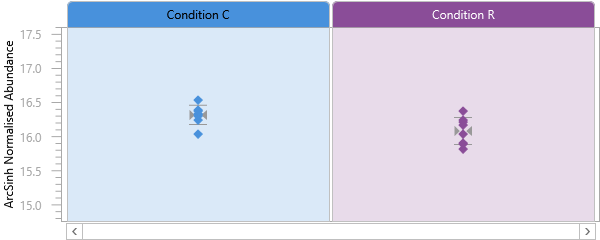

Supplement: Supplementary file 1 [file ijms-20-01156-s001.zip › ID0228_Expression Protein Details_files/protein14_graph.png]

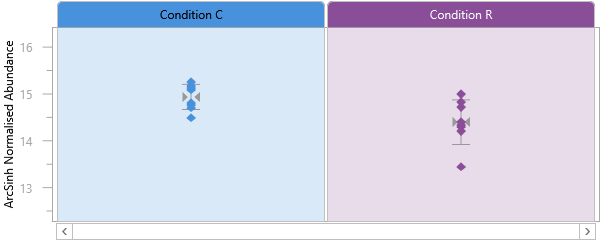

Supplement: Supplementary file 1 [file ijms-20-01156-s001.zip › ID0228_Expression Protein Details_files/protein15_graph.png]

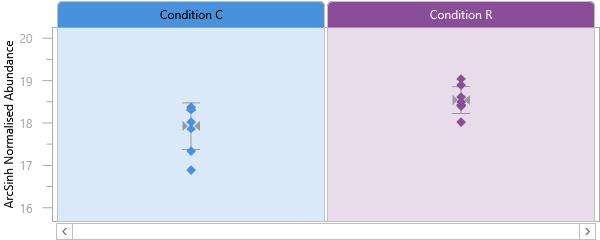

Supplement: Supplementary file 1 [file ijms-20-01156-s001.zip › ID0228_Expression Protein Details_files/protein16_graph.png]

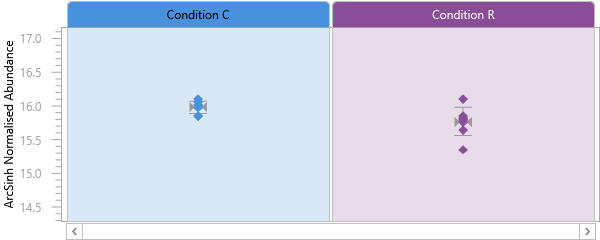

Supplement: Supplementary file 1 [file ijms-20-01156-s001.zip › ID0228_Expression Protein Details_files/protein17_graph.png]

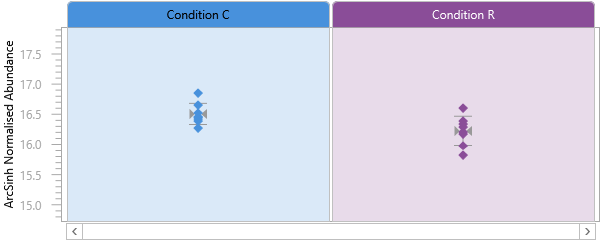

Supplement: Supplementary file 1 [file ijms-20-01156-s001.zip › ID0228_Expression Protein Details_files/protein18_graph.png]

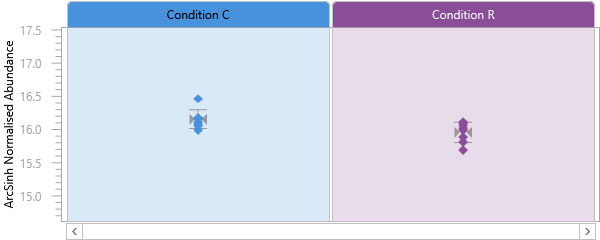

Supplement: Supplementary file 1 [file ijms-20-01156-s001.zip › ID0228_Expression Protein Details_files/protein19_graph.png]

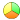

Supplement: Supplementary file 1 [file ijms-20-01156-s001.zip › ID0228_Expression Protein Details_files/protein1Category.png]

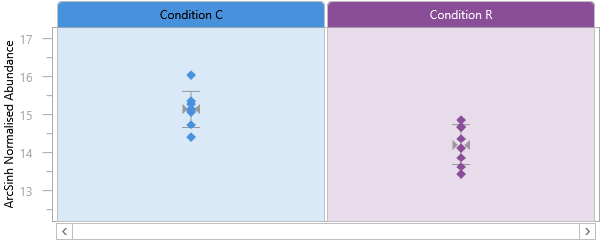

Supplement: Supplementary file 1 [file ijms-20-01156-s001.zip › ID0228_Expression Protein Details_files/protein2_graph.png]

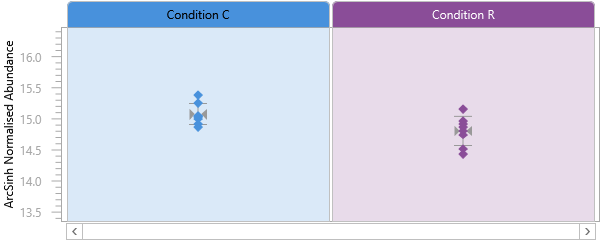

Supplement: Supplementary file 1 [file ijms-20-01156-s001.zip › ID0228_Expression Protein Details_files/protein20_graph.png]

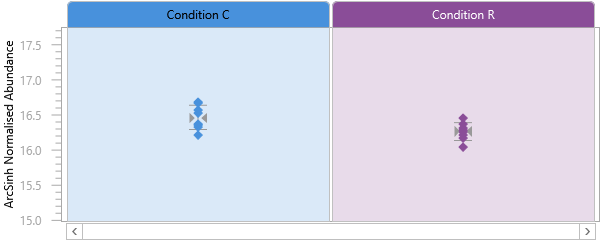

Supplement: Supplementary file 1 [file ijms-20-01156-s001.zip › ID0228_Expression Protein Details_files/protein21_graph.png]

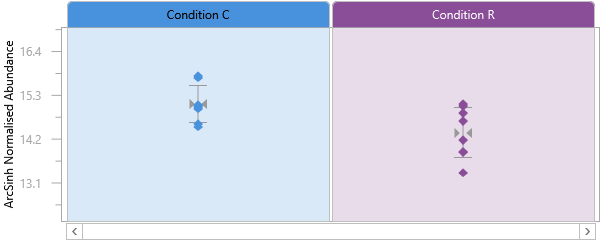

Supplement: Supplementary file 1 [file ijms-20-01156-s001.zip › ID0228_Expression Protein Details_files/protein22_graph.png]

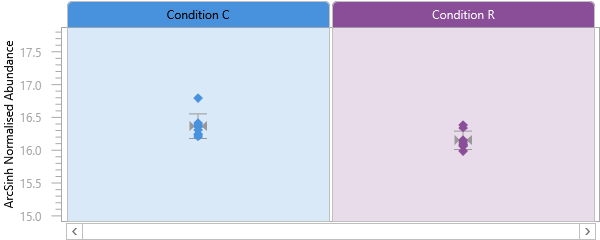

Supplement: Supplementary file 1 [file ijms-20-01156-s001.zip › ID0228_Expression Protein Details_files/protein23_graph.png]

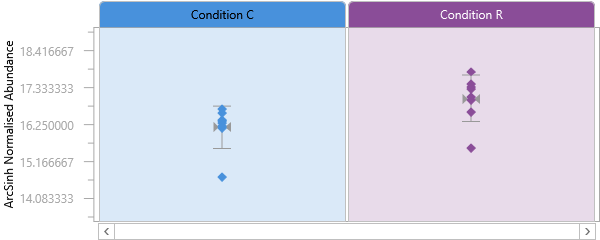

Supplement: Supplementary file 1 [file ijms-20-01156-s001.zip › ID0228_Expression Protein Details_files/protein24_graph.png]

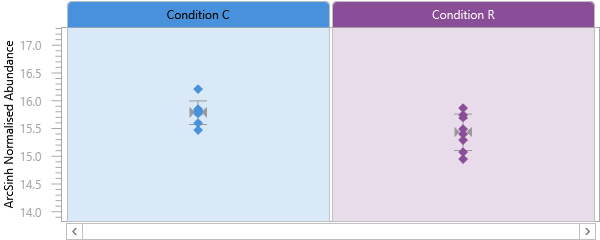

Supplement: Supplementary file 1 [file ijms-20-01156-s001.zip › ID0228_Expression Protein Details_files/protein25_graph.png]

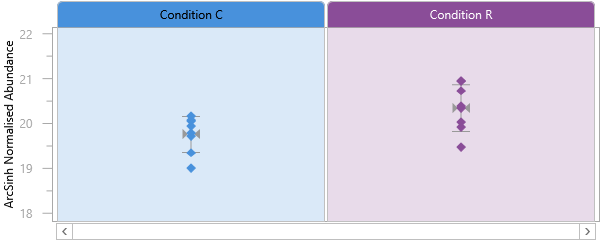

Supplement: Supplementary file 1 [file ijms-20-01156-s001.zip › ID0228_Expression Protein Details_files/protein26_graph.png]

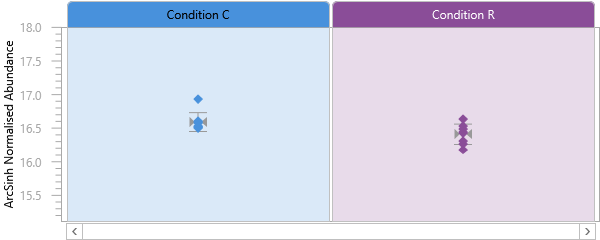

Supplement: Supplementary file 1 [file ijms-20-01156-s001.zip › ID0228_Expression Protein Details_files/protein27_graph.png]

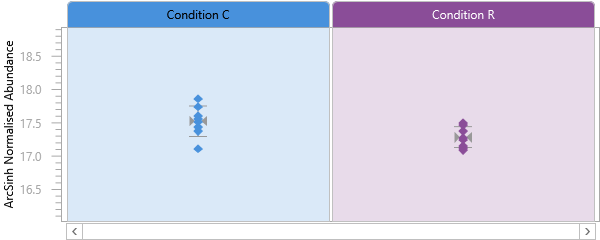

Supplement: Supplementary file 1 [file ijms-20-01156-s001.zip › ID0228_Expression Protein Details_files/protein28_graph.png]

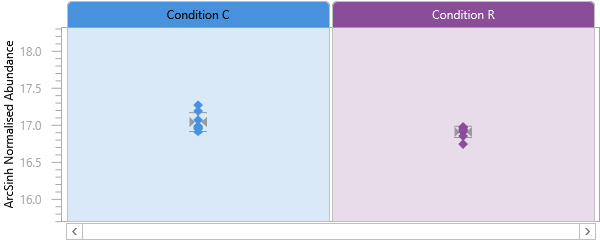

Supplement: Supplementary file 1 [file ijms-20-01156-s001.zip › ID0228_Expression Protein Details_files/protein29_graph.png]

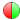

Supplement: Supplementary file 1 [file ijms-20-01156-s001.zip › ID0228_Expression Protein Details_files/protein2Category.png]

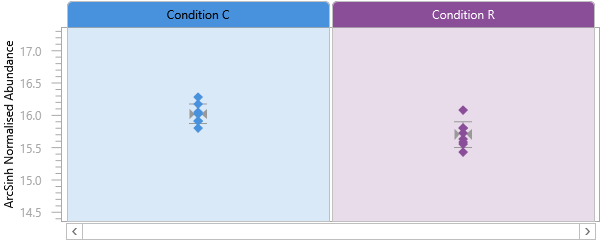

Supplement: Supplementary file 1 [file ijms-20-01156-s001.zip › ID0228_Expression Protein Details_files/protein3_graph.png]

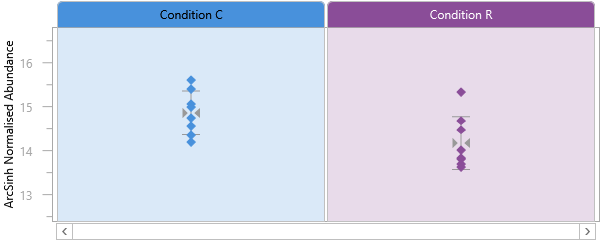

Supplement: Supplementary file 1 [file ijms-20-01156-s001.zip › ID0228_Expression Protein Details_files/protein30_graph.png]

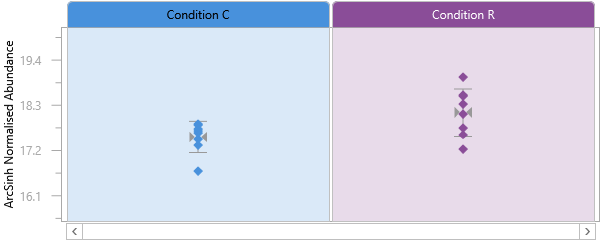

Supplement: Supplementary file 1 [file ijms-20-01156-s001.zip › ID0228_Expression Protein Details_files/protein31_graph.png]

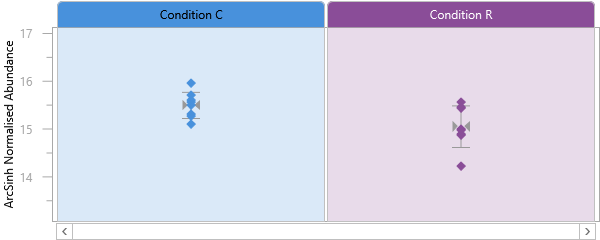

Supplement: Supplementary file 1 [file ijms-20-01156-s001.zip › ID0228_Expression Protein Details_files/protein32_graph.png]

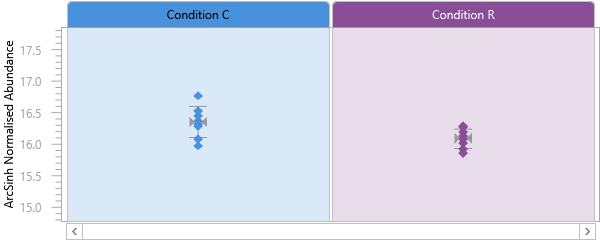

Supplement: Supplementary file 1 [file ijms-20-01156-s001.zip › ID0228_Expression Protein Details_files/protein33_graph.png]

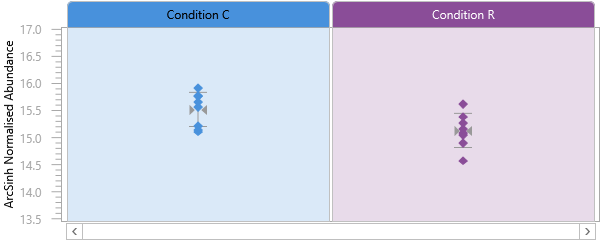

Supplement: Supplementary file 1 [file ijms-20-01156-s001.zip › ID0228_Expression Protein Details_files/protein34_graph.png]

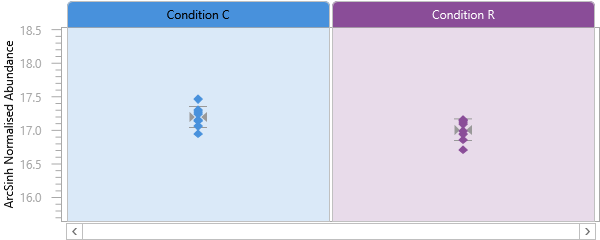

Supplement: Supplementary file 1 [file ijms-20-01156-s001.zip › ID0228_Expression Protein Details_files/protein35_graph.png]

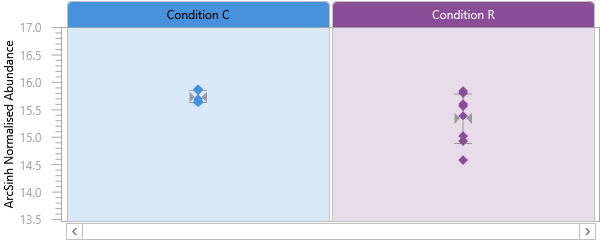

Supplement: Supplementary file 1 [file ijms-20-01156-s001.zip › ID0228_Expression Protein Details_files/protein36_graph.png]

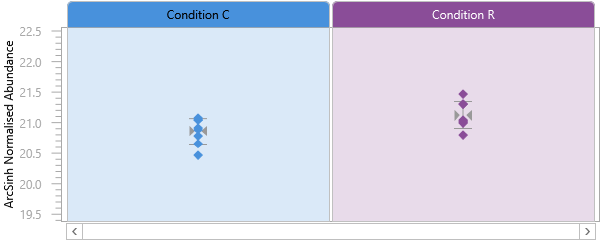

Supplement: Supplementary file 1 [file ijms-20-01156-s001.zip › ID0228_Expression Protein Details_files/protein37_graph.png]

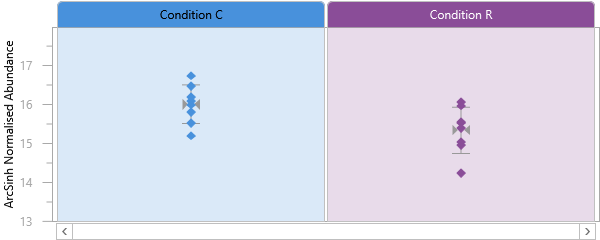

Supplement: Supplementary file 1 [file ijms-20-01156-s001.zip › ID0228_Expression Protein Details_files/protein38_graph.png]

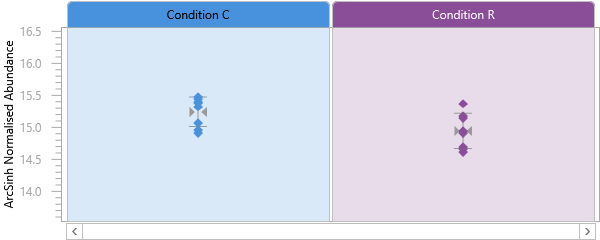

Supplement: Supplementary file 1 [file ijms-20-01156-s001.zip › ID0228_Expression Protein Details_files/protein39_graph.png]

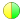

Supplement: Supplementary file 1 [file ijms-20-01156-s001.zip › ID0228_Expression Protein Details_files/protein3Category.png]

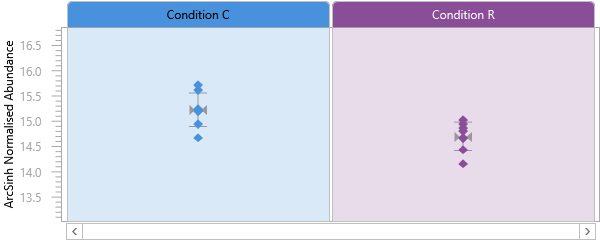

Supplement: Supplementary file 1 [file ijms-20-01156-s001.zip › ID0228_Expression Protein Details_files/protein4_graph.png]

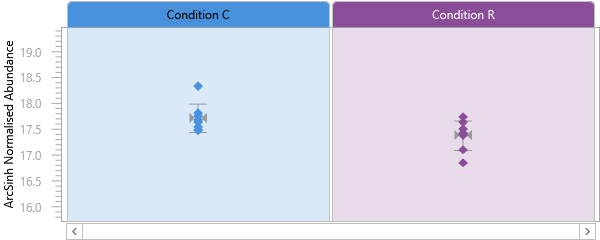

Supplement: Supplementary file 1 [file ijms-20-01156-s001.zip › ID0228_Expression Protein Details_files/protein40_graph.png]

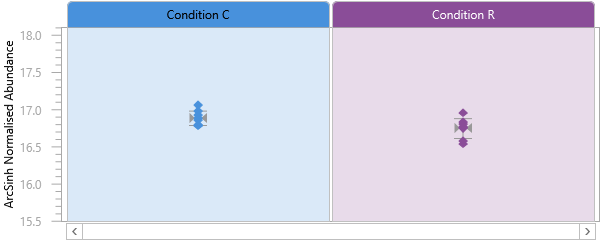

Supplement: Supplementary file 1 [file ijms-20-01156-s001.zip › ID0228_Expression Protein Details_files/protein41_graph.png]

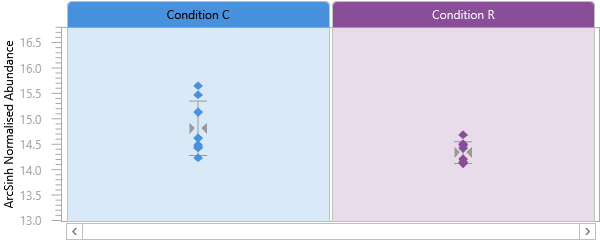

Supplement: Supplementary file 1 [file ijms-20-01156-s001.zip › ID0228_Expression Protein Details_files/protein42_graph.png]

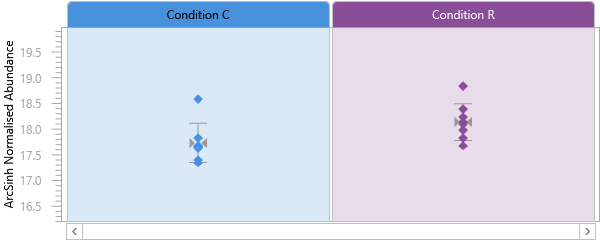

Supplement: Supplementary file 1 [file ijms-20-01156-s001.zip › ID0228_Expression Protein Details_files/protein43_graph.png]

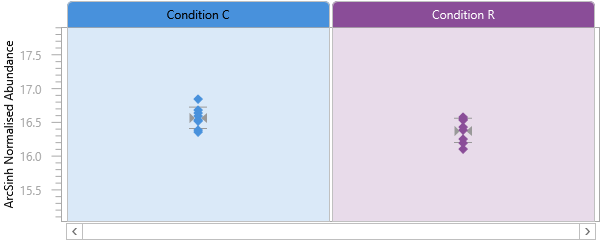

Supplement: Supplementary file 1 [file ijms-20-01156-s001.zip › ID0228_Expression Protein Details_files/protein44_graph.png]

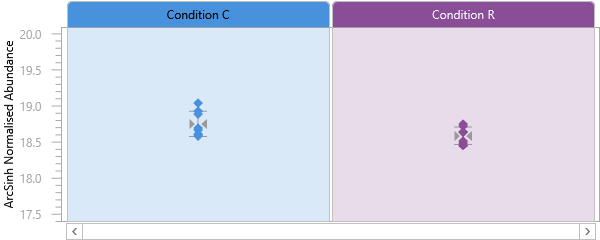

Supplement: Supplementary file 1 [file ijms-20-01156-s001.zip › ID0228_Expression Protein Details_files/protein45_graph.png]

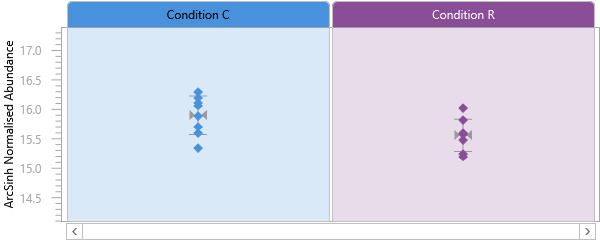

Supplement: Supplementary file 1 [file ijms-20-01156-s001.zip › ID0228_Expression Protein Details_files/protein46_graph.png]

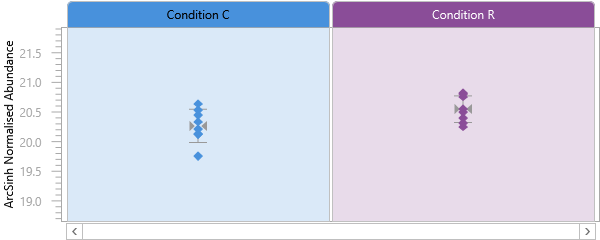

Supplement: Supplementary file 1 [file ijms-20-01156-s001.zip › ID0228_Expression Protein Details_files/protein47_graph.png]

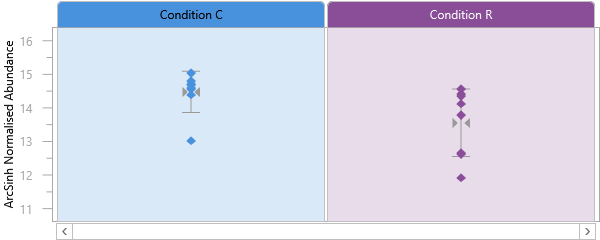

Supplement: Supplementary file 1 [file ijms-20-01156-s001.zip › ID0228_Expression Protein Details_files/protein48_graph.png]

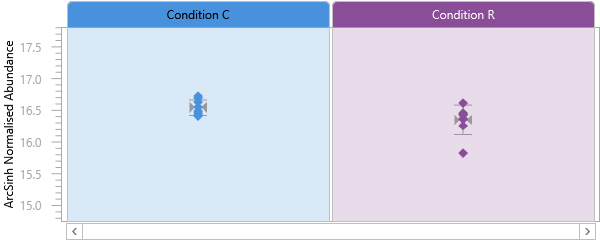

Supplement: Supplementary file 1 [file ijms-20-01156-s001.zip › ID0228_Expression Protein Details_files/protein49_graph.png]

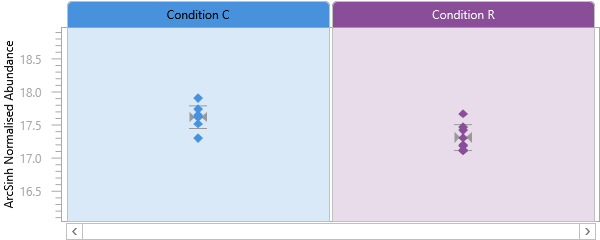

Supplement: Supplementary file 1 [file ijms-20-01156-s001.zip › ID0228_Expression Protein Details_files/protein5_graph.png]

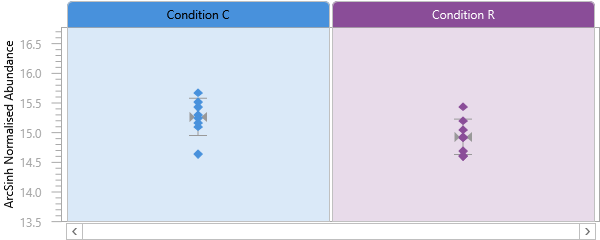

Supplement: Supplementary file 1 [file ijms-20-01156-s001.zip › ID0228_Expression Protein Details_files/protein50_graph.png]

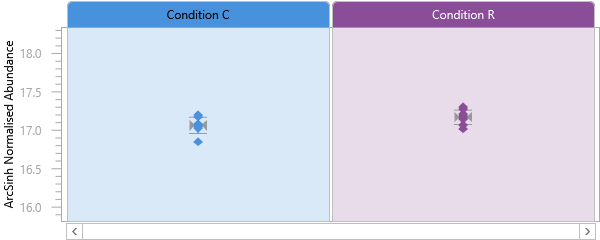

Supplement: Supplementary file 1 [file ijms-20-01156-s001.zip › ID0228_Expression Protein Details_files/protein51_graph.png]

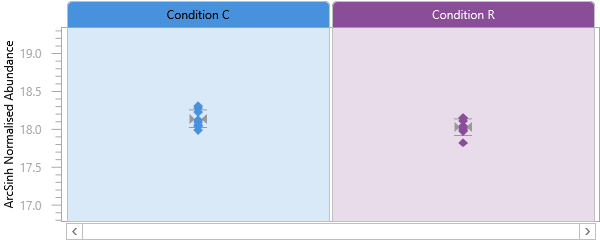

Supplement: Supplementary file 1 [file ijms-20-01156-s001.zip › ID0228_Expression Protein Details_files/protein52_graph.png]

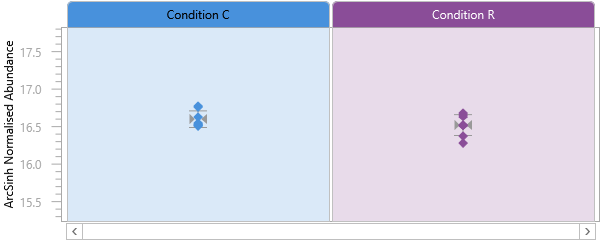

Supplement: Supplementary file 1 [file ijms-20-01156-s001.zip › ID0228_Expression Protein Details_files/protein53_graph.png]

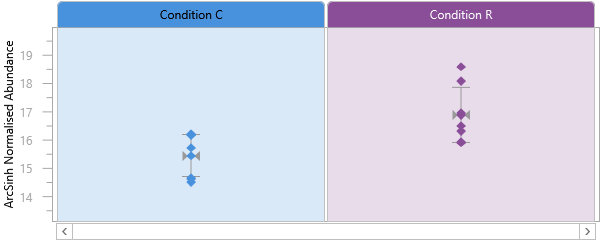

Supplement: Supplementary file 1 [file ijms-20-01156-s001.zip › ID0228_Expression Protein Details_files/protein6_graph.png]

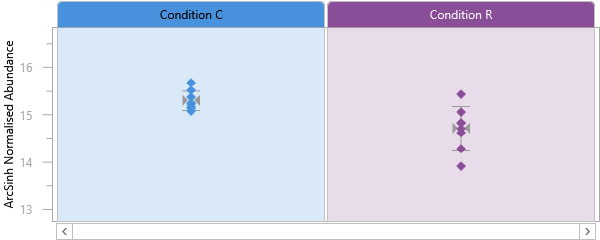

Supplement: Supplementary file 1 [file ijms-20-01156-s001.zip › ID0228_Expression Protein Details_files/protein7_graph.png]

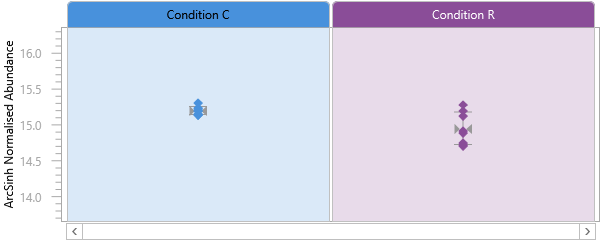

Supplement: Supplementary file 1 [file ijms-20-01156-s001.zip › ID0228_Expression Protein Details_files/protein8_graph.png]

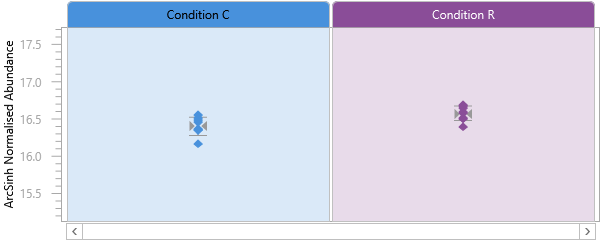

Supplement: Supplementary file 1 [file ijms-20-01156-s001.zip › ID0228_Expression Protein Details_files/protein9_graph.png]

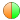

Supplement: Supplementary file 1 [file ijms-20-01156-s001.zip › ID0228_Expression Protein Details_files/protein9Category.png]
